# Pulmonary regurgitant volume is superior to fraction using background-corrected phase contrast MRI in determining the severity of regurgitation in repaired tetralogy of Fallot

**DOI:** 10.1007/s10554-015-0670-6

**Published:** 2015-05-06

**Authors:** Thomas M. Gorter, Joost P. van Melle, Hendrik G. Freling, Tjark Ebels, Beatrijs Bartelds, Petronella G. Pieper, Rolf M. F. Berger, Dirk J. van Veldhuisen, Tineke P. Willems

**Affiliations:** Department of Radiology, University Medical Center Groningen, University of Groningen, Hanzeplein 1, P.O. Box 30.001, 9700 RB Groningen, The Netherlands; Department of Cardiology, University Medical Center Groningen, University of Groningen, Groningen, The Netherlands; Department of Cardiothoracic Surgery, University Medical Center Groningen, University of Groningen, Groningen, The Netherlands; Department of Paediatric Cardiology, University Medical Center Groningen, University of Groningen, Groningen, The Netherlands

**Keywords:** Phase-contrast magnetic resonance imaging, Phase offset errors, Pulmonary regurgitation, Tetralogy of Fallot

## Abstract

In the assessment of pulmonary regurgitation (PR) using phase contrast MRI, phase offset errors affect the accuracy of flow. This study evaluated the use of automated background correction for phase offset in the quantification of PR fraction and volume in patients with repaired tetralogy of Fallot (TOF), and to assess its clinical impact. We retrospectively analyzed 203 cardiac MRI studies, performed on 1.5-T scanner. Pulmonary flow (Q_P_) and systemic flow (Q_S_) was assessed both with and without background correction. Non-corrected and corrected Q_P_ was correlated with Q_S_. PR was correlated with (1) indexed right ventricular end-diastolic volume (RVEDVi) and (2) with differential right and left ventricular stroke volumes (PR_SV_). Both PR fraction and volume showed major change after correction (−43 to +36 % and −13 to +13 ml/m^2^). Corrected Q_P_ and Q_S_ were stronger correlated with each other than non-corrected Q_P_ and Q_S_ [r = 0.78 vs. 0.73 (*p* < 0.001)]. Both PR fraction and volume were stronger correlated with RVEDVi, compared to their non-corrected counterparts (*p* < 0.001). PR volume was stronger correlated with RVEDVi, compared to PR fraction [r = 0.74 vs. 0.69 (*p* < 0.001)]. When patients were divided according to PR severity, 12 % of patients reclassified after correction. Background correction for phase offset significantly changed the quantification of PR. Non-corrected assessment of PR may result in the misclassification of patients. Our data suggest that the use of PR volume is favourable in the follow-up of patients with repaired TOF.

**Electronic supplementary material:**

The online version of this article (doi:10.1007/s10554-015-0670-6) contains supplementary material, which is available to authorized users.

## Introduction

In patients with repaired tetralogy of Fallot (TOF), longstanding pulmonary regurgitation (PR) is the most important cause of right ventricular (RV) dilatation and subsequent failure. The degree of PR may guide clinicians in determining timing for re-intervention [1, 2]. Therefore, correct assessment of PR in these patients is essential.

For the quantification of PR, phase contrast cardiovascular magnetic resonance imaging (MRI) is the accepted and recommended method [3–5]. However, the assessment of PR using MRI is also accompanied by limitations and uncertainties. In phase contrast MRI, the velocity of blood flow in the main pulmonary artery (MPA) is derived from the rotation (i.e. phase shift) of moving spins along a magnetic field gradient. The amount of phase shift is proportional to the velocity of these moving spins [6–8]. However, phase offset errors induced by non-compensated eddy current fields and concomitant gradient terms cause velocity offsets. These widely recognized errors affect the accuracy of flow measurements [6, 9].

Over the years, several techniques have been developed to correct for offset errors. For example, observer defined stationary tissue close to the vessel of interest and stationary phantom derived corrections improve flow measurements [10–14]. In cardiac MRI however, stationary tissue is rarely nearby the vessel of interest. Although phantom correction is reliable and is used as the reference standard, offset errors often vary with different slice orientation and position. Especially for congenital heart diseases this phantom correction may therefore be time-consuming, because clinical assessment of these patients generally include multiple phase contrast sequences. Therefore, post-processing image correction, based on phase offset elimination across regions of signal from static tissues, has been developed [15–17]. This automated background correction method is promising, yet the clinical consequences are unknown. Further studies in validating these methods in clinical practice are therefore recommended [18].

Phase contrast measurements of the MPA include forward volume and regurgitant volume. PR fraction is the ratio of regurgitant and forward volume. Both regurgitant volume and fraction represent PR, yet they are poorly interchangeable [19]. Because phase offset errors affect both forward and regurgitant volume, we hypothesized that the impact of these errors is larger on regurgitant fraction than on regurgitant volume.

The purpose of this study is to evaluate the use of automated background correction in the quantification of PR in patients with repaired TOF and to assess the impact on clinical decision making.

## Materials and methods

We retrospectively assessed all patients with repaired TOF who underwent routine cardiac MRI between January 2007 and March 2013, including phase contrast acquisition of the MPA. MRI studies with velocity aliasing or visible MRI artefacts in the MPA field were excluded. Baseline variables included demographics, surgical history (i.e. type of initial correction and type of any PVR prior to MRI), echocardiographic data within 1 year of the MRI study [i.e. pulmonary valve (PV) peak gradient, presence of ≥moderate tricuspid regurgitation (TR) or any residual atrial or ventricular septal defect]. PR duration was set from the date of transannular patch (TAP) insertion until date of MRI or from the first time point of ≥moderate PR in patients without TAP, assessed with echocardiography. This study complies with the Declaration of Helsinki. The local Institutional Review Board had no objections to the use and publication of the acquired patient data. Because of the retrospective character of the study, the need for individual informed consent was waived.

### Cardiac magnetic resonance imaging

MRI protocols and acquisitions used for the assessment of ventricular volumes and flow have been previously published by our group in detail [20, 21]. In brief, studies were performed using a 1.5-T MR scanner [Magnetom Avanto (or SonataVision), Siemens, Erlangen, Germany]. After single-shot localizer images, short-axis cine loop images with breath holding in expiration were acquired using a retrospectively gated balanced steady-state free precession sequence. Two-dimensional velocity encoded MRI flow measurements, perpendicular and directly cranial to the pulmonary and aortic valve, were performed using 2-D gradient echo Fast Low Angle SHot (FLASH), acquired during normal respiration with retrospective cardiac gating.

### Cardiac magnetic resonance imaging analysis

The endo- and epicardial contours of the left and right ventricle were traced manually on end-diastolic and end-systolic phases using QMass 7.6 (Medis, Leiden, The Netherlands). On the most basal slice, the right atrium and the pulmonary artery were excluded. For the RV, papillary muscle and trabeculae were excluded from RV blood volume by using semi-automatic threshold-based segmentation software, based on previous description [20]. End-diastolic and end-systolic volumes were then automatically calculated. Stroke volume (SV) was defined as end-diastolic volume minus end-systolic volume and ejection fraction as stroke volume divided by end-diastolic volume.

Analyses of MPA and aortic flow were performed according to current recommendations [18], using QFlow 5.6 (Medis, Leiden, The Netherlands). MPA and aortic contours were generated semi-automatically on the standard magnitude images and were manually adjusted for each phase image.

As background offset correction was an integral part of QFlow 5.6, post-processing background correction and image reconstruction were performed using the same images and contours that were used for the previously derived non-corrected series. Both non-corrected and corrected pulmonary flow (Q_P_), systemic flow (Q_S_) and forward and regurgitant volume were documented. Visualisation of automated background correction for phase offset errors is illustrated as Supplementary material (Figure S1).

Absolute volumetric measurements were indexed for body surface area (BSA) using the calculation of Haycock [22]. PR fraction was calculated as the ratio between regurgitant and forward volume and graded as insignificant or mild (<25 %), moderate (25–40 %) and severe (≥40 %) [5, 23].

In the absence of a golden standard for MPA flow, the background correction method was tested using three different approaches. First, after exclusion of patients with intracardiac shunts (n = 12), both non-corrected and corrected Q_P_ were correlated with non-corrected and corrected Q_S_. Second, regurgitant volume was calculated by the difference between LV and RV stroke volume (PR_SV_ = RVSV–LVSV) [19]. Both non-corrected and corrected PR volume were correlated with PR_SV_. Third, given the well-known relationship between PR and RV dilatation, also correlations were made between non-corrected and corrected PR fraction and volume, and indexed RVEDV (RVEDVi).

All phase contrast and ventricular volume measurements were performed by a single experienced observer who was blinded for the surgical history (TG). Intra-observer reliability for the primary outcome measurements was assessed using twenty random MRI studies. Inter-observer reliability was assessed by two observers (an experienced laboratory technician and TG) using again twenty random studies.

### Statistical analysis

Data were reported as mean ± SD for normally distributed data, median (interquartile range) for skewed distributed data or n (%) for dichotomous variables. Difference between non-corrected and corrected measurements were performed using Wilcoxon signed-rank tests. Bland–Altman analyses were performed to analyze the agreement between both methods. Differences in baseline between subgroups were analyzed using independent samples *t* tests or Mann–Whitney *U* test, according to distribution. Correlation between normally distributed continuous variables were done using Pearson’s correlation and correlation between not normally distributed variables were performed with Spearman’s rank correlation.

In a univariate regression model, correlations with RVEDVi were made between corrected PR fraction and volume, late diastolic forward volume (for the detection of restrictive physiology), PR duration, PV peak gradient, presence of ≥moderate TR or any residual atrial or ventricular septal defect. Variables with a *p* value <0.1 were added to the multivariate model. The multivariate model was tested for collinearity.

Reclassification analysis was performed, according to the amount of patients that changed PR fraction class (i.e. ≥1 class) after background correction.

A substantial part of the study population consist of multiple MRI studies for single patients. In order to correct for potential bias, sensitivity analysis consisted of re-analyses with exclusion of these repeated MRI’s. In addition, the same analyses were performed in a subset of cases without inserted mechanical valves. Correlations between PR measurements and RVEDVi (with inclusion of trabeculae and papillary muscles) were also performed.

Intra-observer and inter-observer variability was assessed using Two-way mixed Intraclass Correlation Coefficient (ICC).

Statistical significance was considered achieved at a *p*-value <0.05. Statistical analyses were performed using SPSS statistical software (Version 20, 2011).

## Results

In total 246 MRI studies were identified. Forty-three studies were excluded due to wraparound (n = 30) or mechanical artefacts (n = 2) in the MPA field, aliasing (n = 3), claustrophobia (n = 6) or poor ECG-triggered-cine (n = 2). The final study population consisted of 203 MRI studies in 134 unique patients. Baseline demographic and surgical characteristics of the study population are outlined in Table 1. Thirty-four children [mean 12.7 ± 3.1 (range 3.5–17) years] were included (17 %). Ventricular volume and function data are also depicted in Table 1. In 10 MRI studies, reliable volumetric measurements in the short-axis acquisition were not possible due to poor ECG-triggered cine or artefacts. The median interval between MRI and echocardiographic assessment was 42 (21–84) days.

**Table 1:** Baseline characteristics of the total study population (n = 203)

*Demographics*	
Sex, male	111 (54.7 %)
BSA (m^2^)	1.82 ± 0.31
Age at MRI study (years)	28.2 (20.1–36.9)
*Surgical history*	
Age at TOF repair (years)	1.8 (1.1–5.4)
Type of TOF repair	
TAP	133 (65.5 %)
No-TAP	54 (26.6 %)
Conduit	8 (3.9 %)
Unknown	8 (3.9 %)
PVR prior MRI	45 (22.2 %)
PVR type, mechanical	16 (35.6 %)
PVR type, bio	29 (64.4 %)
Bio type	
Homograft	12 (41.4 %)
Contegra conduit	3 (10.3 %)
Bioprosthesis	14 (48.3 %)
*MRI*	
Time from TOF repair to MRI (years)	26.0 (18.1–31.2)
RVEDVi (ml/m^2^)	127 ± 36
RVEF (%)	49 ± 8
LVEDVi (ml/m^2^)	77 ± 16
LVEF (%)	54 ± 7
*Echocardiography*	
PR duration (years)	15.3 (9.9–25.7)
PV peak gradient (mmHg)	20.9 (13.5–30.3)
TR (≥moderate)	19 (9.4 %)
Residual ASD	2 (1.0 %)
Residual VSD	10 (4.9 %)

Data is reported as mean ± SD, median (interquartile range) or n (%)

*BSA* body surface area, *LVEDVi* indexed left ventricular end-diastolic volume, *LVEF* left ventricular ejection fraction, *MRI* magnetic resonance imaging, *PR* pulmonary regurgitation, *PV* pulmonary valve, *PVR* pulmonary valve replacement, *RVEDVi* indexed right ventricular end-diastolic volume, *RVEF* right ventricular ejection fraction, *TAP* transannular patch, *TOF* tetralogy of Fallot, *TR* tricuspid regurgitation

### Flow analyses with background correction

**Fig. 1:**
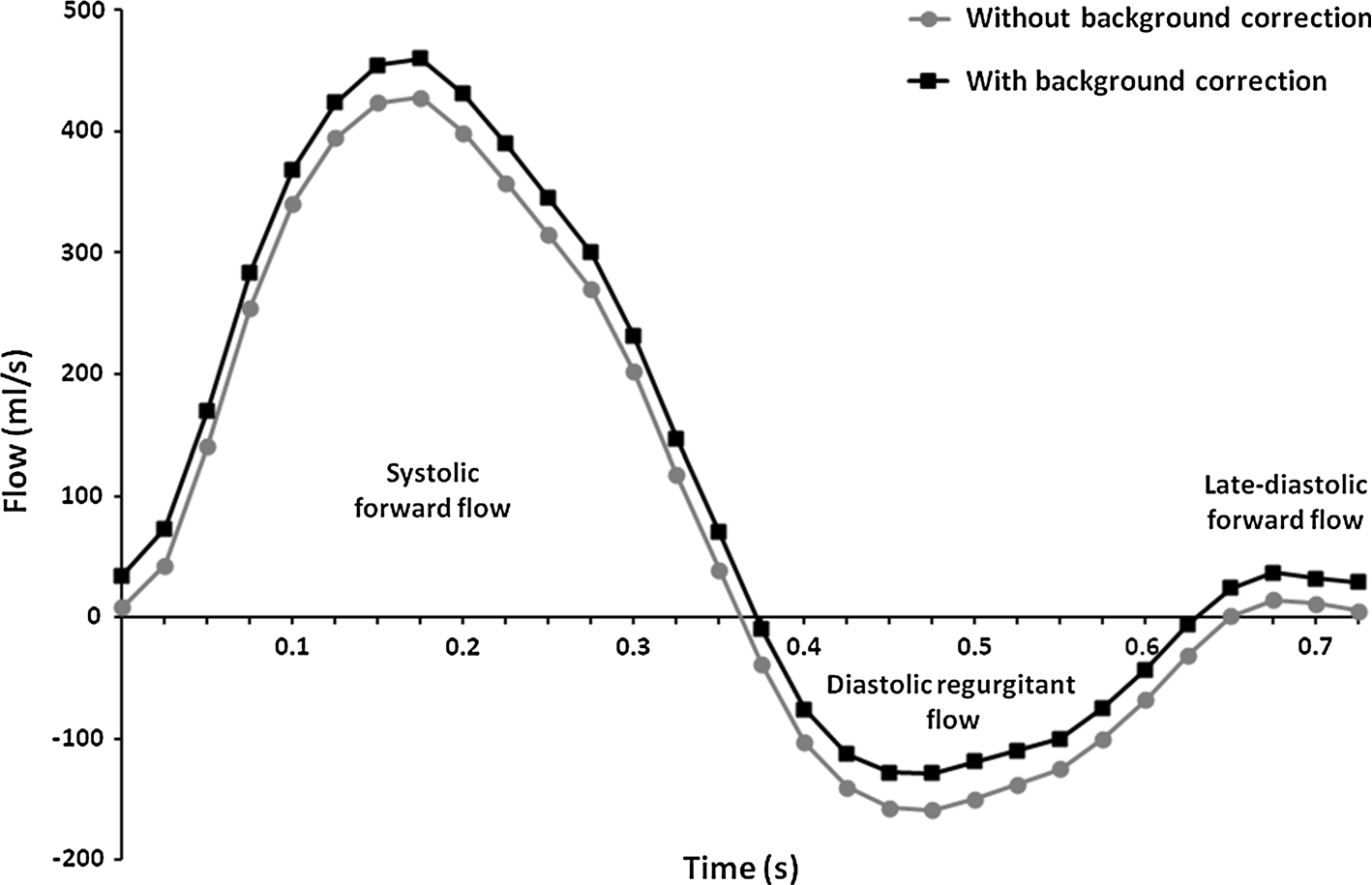
Flow velocity curve. MPA flow-velocity curve of a patient, without (*round dots*) and with (*square dots*) background correction. Area under the curve (i.e. time–velocity integral) represents the systolic forward volume, diastolic regurgitant volume and late-diastolic forward volume. In this example, PR fraction changed from 32 to 21 %, caused by change in both forward and regurgitant volume

The non-corrected flow measurements of the MPA and the average change after background correction are outlined in Table 2. Overall, there is a large variation of change after correction. The change in PR fraction ranged from −35 to +44 % and for PR volume this was −13 to +13 ml/m^2^. Figure 1 is an example of a flow-velocity curve of a patient with PR, both with and without background correction.

**Table 2:** The effects of background correction on flow measurements of the main pulmonary artery

	Non-corrected measurements	Change after background correction	*p* value
PFV (ml/m^2^)	57.7 (47.8–69.8)	+0.8 (−18.3 to 11.0)	0.001
PR volume (ml/m^2^)	16.9 (3.3–27.7)	−0.7 (−13.3 to 13.4)	<0.001
PR fraction (%)	31.0 (7.7–42.0)	−1.8 (−35.5 to 43.8)	<0.001
Q_P_ (l/min)	5.3 (4.2–6.5)	+0.2 (−4.3 to 2.8)	<0.001

Data is reported as median (interquartile range) for non-corrected measurements and mean (range) for change after background correction

*Q*_*P*_ flow in the main pulmonary artery, *PFV* pulmonary forward volume, *PR* pulmonary regurgitation

**Fig. 2:**
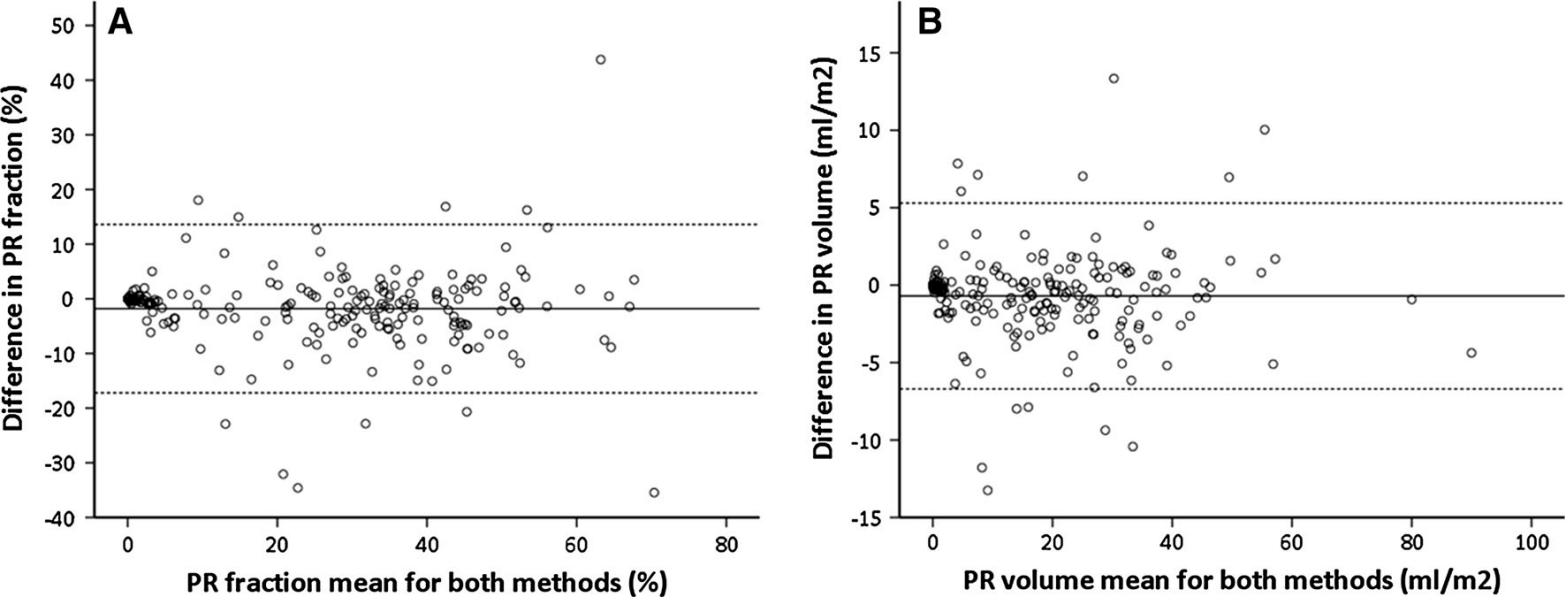
Bland–Altman plots. **a** *Scatter plot* show the mean in PR fraction of both measurements, compared to the difference between both methods. **b** *Scatter plot* show the mean in PR volume of both measurements, compared to the difference between both methods. The *solid lines* represent the mean of the difference. The *dotted lines* represent two standard deviations of that mean in each direction

Differences in PR fraction and volume between non-corrected and corrected measurements showed small bias and a large variation based on the magnitude of PR fraction and volume (Fig. 2a, b). Eleven patients (5 %) had a change in PR fraction ≥2-SD. For PR volume this change accounts for 13 patients (6 %).

**Fig. 3:**
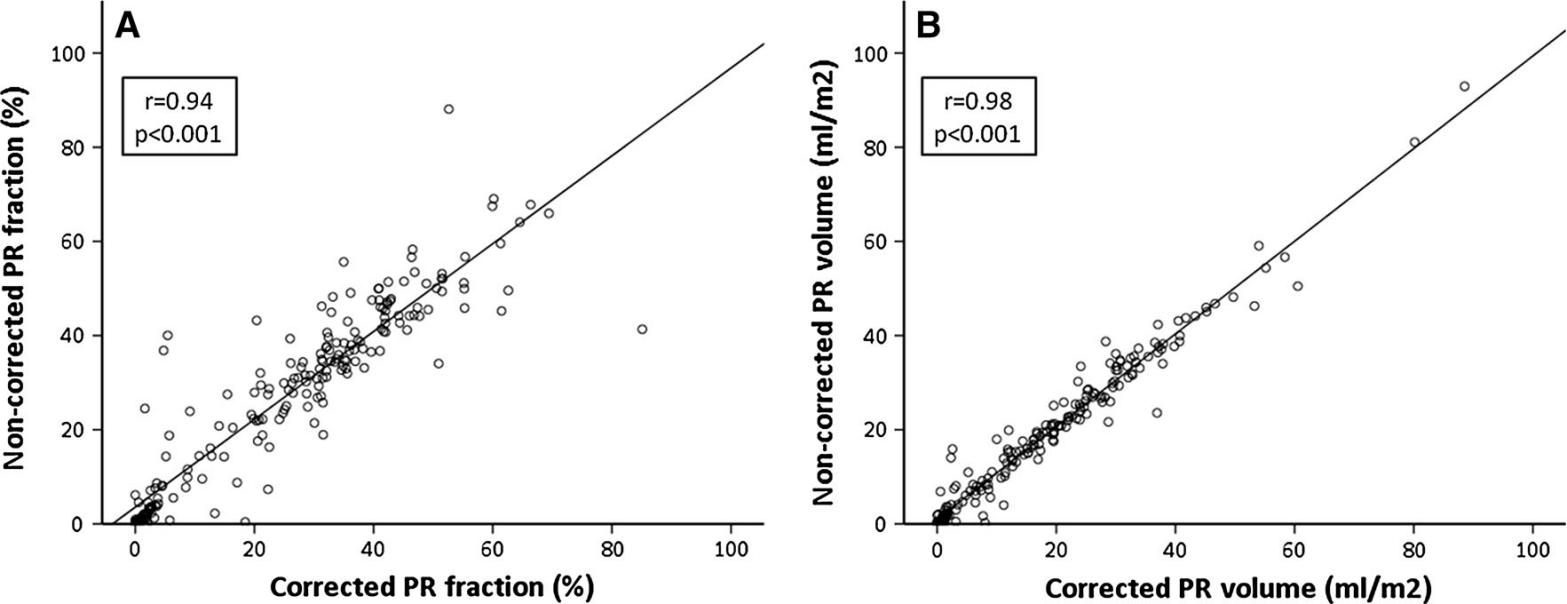
Comparison between non-corrected and corrected measurements. **a** *Scatter plot* demonstrating the correlation between non-corrected and corrected PR fraction. **b** Demonstrating the correlation between non-corrected and corrected PR volume

The relationship between non-corrected and corrected regurgitant measurements is depicted in Fig. 3a, b. Correlation between non-corrected and corrected PR volume was stronger, compared to non-corrected and corrected PR fraction [r = 0.98 vs. 0.94 (*p* < 0.001)]. The correlation between non-corrected pulmonary forward volume with corrected pulmonary forward volume was 0.96, *p* < 0.001.

In the absence of intracardiac shunts, Q_P_ equals Q_S_ minus flow to the coronary sinus. Corrected Q_P_ and Q_S_ were stronger correlated with each other than the non-corrected measurements [0.78 vs. 0.73, respectively (*p* < 0.001)]. There was no difference in the correlation between non-corrected and corrected PR volume, and PR_SV_ (both 0.79).

### Correlations with right ventricular dilatation

Both non-corrected and corrected regurgitant measurements were correlated with RVEDVi. Correlations between corrected regurgitant measurements and RVEDVi were stronger, compared to their non-corrected counterparts [PR fraction: 0.69 vs. 0.63 (*p* < 0.001), respectively; PR volume 0.74 vs. 0.72 (*p* < 0.001), respectively].

**Fig. 4:**
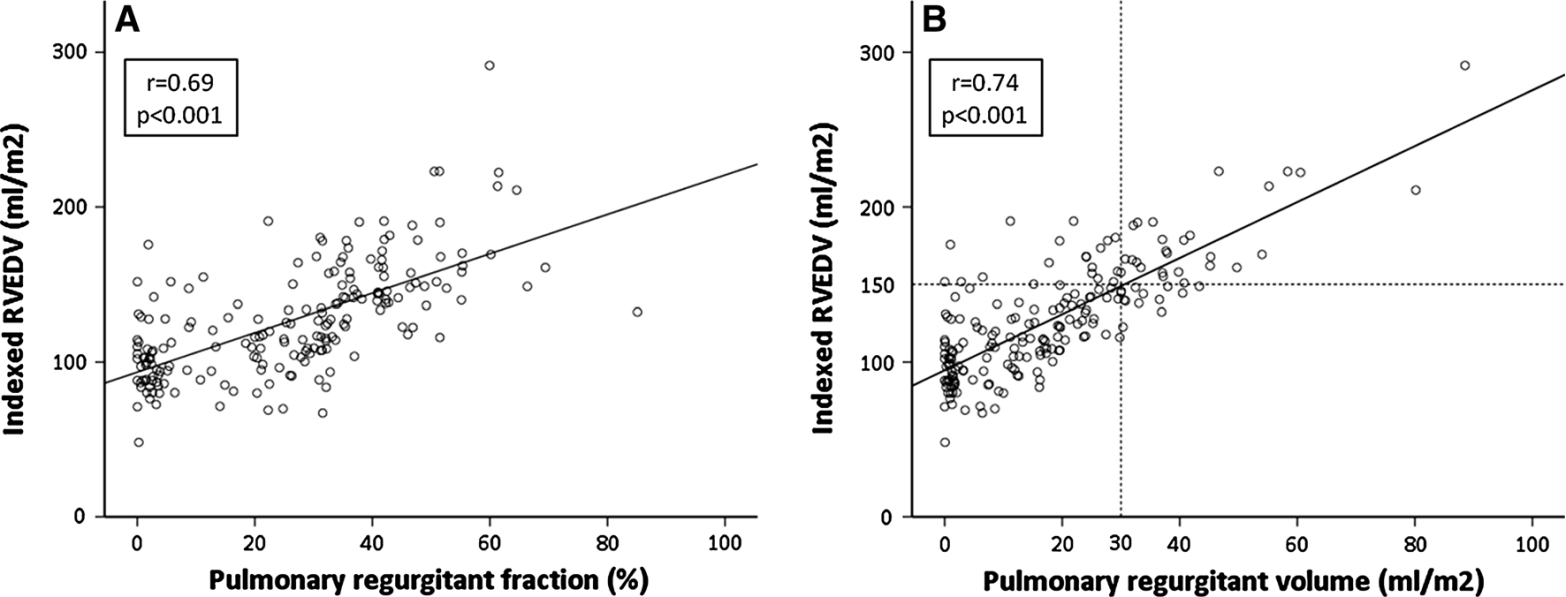
Comparison with indexed RVEDV. **a** *Scatter plot* demonstrating the correlation between PR fraction and RVEDVi. **b** Demonstrating the correlation between PR volume and RVEDVi. RVEDVi of 150 ml/m^2^ corresponds with 30 ml/m^2^ PR volume

In the univariate model, PR fraction, PR volume, late diastolic forward volume and PV peak gradient were significant correlated with RVEDVi (Table 3). In the multivariate model, corrected PR volume remained independently associated with RVEDVi (*p* < 0.001). PR volume was stronger correlated to RVEDVi than PR fraction, as seen in Fig. 4a, b (r = 0.74 vs. 0.69, respectively). RVEDVi of 150 ml/m^2^ corresponds with 30 ml/m^2^ PR volume, as seen in Fig. 4b.

**Table 3:** Linear regression model of RVEDVi

	*Univariate*		*Multivariate*	
	β	*p*-value	β	*p*-value
Corrected PR fraction	0.652*	<0.001		
Corrected PR volume	0.791*	<0.001	0.781	<0.001
Late diastolic forward volume	0.463	<0.001	−0.020	0.984
PR duration	−0.091	0.273		
PV peak gradient	−0.251	<0.001	−0.038	0.413
TR (>moderate)	−0.056	0.440		
Residual VSD/ASD	−0.004	0.960		

*ASD* atrial septal defect, *PR* pulmonary regurgitation, *PV* pulmonary valve, *RVEDVi* indexed right ventricular end diastolic volume, *VSD* ventricular septal defect

* Only PR volume was added to the multivariate analysis due to collinearity with PR fraction

### Sensitivity analysis

After exclusion of repeated MRIs and/or inserted mechanical valves prior to MRI (n = 77), corrected Q_P_ and Q_S_ remained stronger correlated with each other than non-corrected measurements (0.78 vs. 0.68, respectively). In addition, the corrected measurements of PR fraction and volume remained significant predictors of RVEDVi, in contrast to their non-corrected counterparts (*p* < 0.001 and 0.001, respectively).

When both methods of RVEDVi measurements (i.e. inclusion and exclusion of trabeculae and papillary muscle) were correlated with corrected PR fraction and volume, only the exclusion method remained correlated with PR fraction and volume (*p* = 0.030 and 0.009, respectively).

### Reclassification analysis

Table 4 shows reclassification according to the severity of PR. At baseline, 83 patients (41 %) had insignificant or mild PR, 61 moderate (30 %) and 59 (29 %) severe. After correction, the reclassification rate was 12.3 %. In six patients (3 %) the amount of PR was underestimated and in 19 patients (9 %) PR severity was overrated. Fourteen patients (7 %), changed across 25 % PR fraction. Eight of the 19 patients in whom PR was overrated, received a PVR after MRI. Only three of these patients had an RVEDVi above 150 ml/m^2^. None of the six patients in whom PR was underestimated received PVR after MRI. Two of these patients had an RVEDVi above 150 ml/m^2^.

**Table 4:** Reclassification into different pulmonary regurgitation severity classes according to background corrected flow-measurements (n = 203)

Corrected PR fraction	Non-corrected PR fraction			Total
	<25 %	25–40 %	≥40 %	
<25 %	79	8	2	89
25–40 %	4	51	9	64
≥40 %	0	2	48	50
Total	83	61	59	178

Data is reported as n. PR pulmonary regurgitation. Reclassification rate is 12.3 % [(203–178)/203 * 100]

When patients with inserted mechanical valves prior MRI assessment were excluded in this analysis (n = 16), the overall reclassification rate was 13.4 % and the reclassification across 25 % PR fraction was 7.5 %.

### Intra-observer and inter-observer variability

Intraclass correlation for intra-observer variability showed strong correlation for both non-corrected and corrected PR fraction [ICC = 1.0 (95 % CI 0.99–1.0); ICC = 0.99 (95 % CI 0.97–1.0), respectively]. For inter-observer variability the correlation for non-corrected PR fraction was 0.93 (95 % CI 0.82–0.97) and for corrected PR fraction 0.95 (95 % CI 0.87–0.98). *P*-values for all correlations were <0.001.

## Discussion

There are three major findings of the current study. First, background correction resulted in significant change in PR fraction and volume. Second, the effect of background correction was more evident on PR fraction than PR volume. Third, this study showed that when background correction is used, 12 % of Fallot patients were reclassified according to their PR severity.

PVR is recommended when symptoms are unequivocally due to severe PR [1]. Timing of PVR in asymptomatic patients however, remains a matter of debate. Yet 25 % regurgitation fraction is considered an important cut-off in PR severity [23]. Hence, a correct quantification of PR, in order to assess timing for re-intervention, is key.

Recently, automated background correction was assessed in a heterogeneous population of patients referred for phase contrast MRI, including 15 TOF patients. Background correction improved the quantification of flow in these patients [17]. However, clinical implications for a homogenous cohort of patients were lacking.

In the current study, we measured significant changes in PR fraction and volume after using the background correction approach. In experimental settings, measured volumes can be validated using flow probes to assess forward flow in large vessels. Such an approach is not feasible in patients. Yet theoretically, aortic flow and RVEDVi should be strongly related to pulmonary flow and pulmonary regurgitation, respectively. Indeed in our study, we showed that after background correction, correlations between both parameters improved.

The next question is to what the clinical implications are of these differences. After background correction, we demonstrated an overall reclassification rate of 12 %, according to the severity of PR.

In the majority of these patients (76 %), the amount of PR was overrated. Hence possibly these patients would have been incorrectly referred for intervention. In the remaining part (24 %), the amount of PR was underestimated, which could have lead to erroneously withholding these patients an intervention. This is particularly important since the indications for intervention or PR are shifting towards earlier repair before clinical signs and symptoms succeed.

As a consequence, all patients in whom PVR is considered a therapeutic option, may benefit from automated background correction. Because the observed change in PR after background correction is in either direction, difference in PR measurements between two consecutive MRI studies may be the result of difference in impact of offset errors, rather than change in PR severity. Because of this large variation after correction, we recommend the consistent use of automated background correction in these patients.

Interestingly, we observed a stronger correlation between non-corrected and corrected PR volume and pulmonary forward volume than between non-corrected and corrected PR fraction. PR fraction is the result of the ratio of regurgitant to forward volume. Because phase offset errors affect both forward and regurgitant volume, any effects on these flow volumes are amplified by the PR fraction calculation. Previously, the value of regurgitant volume above fraction, in relation to RV dilatation, was demonstrated in adults with repaired TOF [19, 24]. In our large cohort of both children and adults, we also showed a stronger relationship between regurgitant volume and RVEDV, compared to regurgitant fraction. Regurgitant volume seems to better represent the physiological consequences of regurgitation. Given that PR volume is also less influenced by background errors, the use of regurgitant volume seems favourable in the follow-up of these patients.

An RVEDV cut-off value of 150 ml/m^2^ is considered an important item in the indication for PVR in patients with ≥25 % PR fraction [23]. We observed that this RVEDV value has a reasonable correlation with a corrected PR volume of 30 ml/m^2^. Further studies are needed to investigate whether such a cut-off value, based on absolute regurgitation volume, may serve as potential threshold in the discussion on timing for re-intervention in repaired tetralogy of Fallot.

### Limitations

Our study has some limitations that merit emphasis. First, automated background corrections for phase offset errors were performed using an MRI scanner of one single vendor. The results of this study are not automatically applicable on flow data from scanners of other vendors. There is however widespread clinical consensus that phase offset errors are of different importance among scanners of different vendors. Furthermore, a gold standard for the assessment of MPA flow was lacking. However, aortic flow, differential ventricular stroke volume and RVEDV were used as reference. There still remained a gap in the correlation between corrected Q_P_ and Q_S_, independent of objected residual shunt lesions. We were not able to quantify flow to the coronary sinus. Furthermore, there may still be non-objected shunt lesions or remaining measurement errors between aortic and pulmonary flow. Finally, although the correction method is implemented in the analysis software, human involvement in background correction cannot be excluded. Yet, both inter-observer and intra-observer variability showed strong correlations.

## Conclusions

Cardiovascular MRI is the cornerstone in clinical decision making in patients with repaired TOF and residual PR. We showed that automated background correction significantly changed the measured PR and that phase offset errors have less influence on PR volume than on PR fraction. PR volume is also stronger related to RV volume, compared to PR fraction. Therefore, PR volume seems favourable in the follow-up of these patients. Non-corrected flow measurements may result in the misclassification of patients according to the severity of PR. Further studies are needed to validate background correction in a multicenter setting and across different vendors.

## Electronic supplementary material

Supplementary material 1 (DOC 661 kb)
