# Supplementary material for: Pulmonary regurgitant volume is superior to fraction using background-corrected phase contrast MRI in determining the severity of regurgitation in repaired tetralogy of Fallot
Source: Int J Cardiovasc Imaging. 2015 May 6;31(6):1169–77. doi: 10.1007/s10554-015-0670-6 (PMC4486784; doi:10.1007/s10554-015-0670-6)

**SUPPLEMENTARY MATERIAL**

**Figure S1: Flow offset in static tissue.** In velocity-encoded phase contrast images, three types of flow regions can be found: 1) air with random phase (e.g. lungs), 2) vessels with flow and 3) static tissue. In theory, the flow in static tissues equals zero. In practice, errors occur, causing flow offsets that vary slowly over the image. Automated background correction for phase contrast estimates and corrects this offset by measuring the flow offset in static tissue and interpolating it to non-static tissue. Thereby it provides a correction to the flow offset in vessels, regardless of where it is located in the image. In this example, the offset field spreads from approximately -8 cm/s (red) to +4 cm/s (blue).


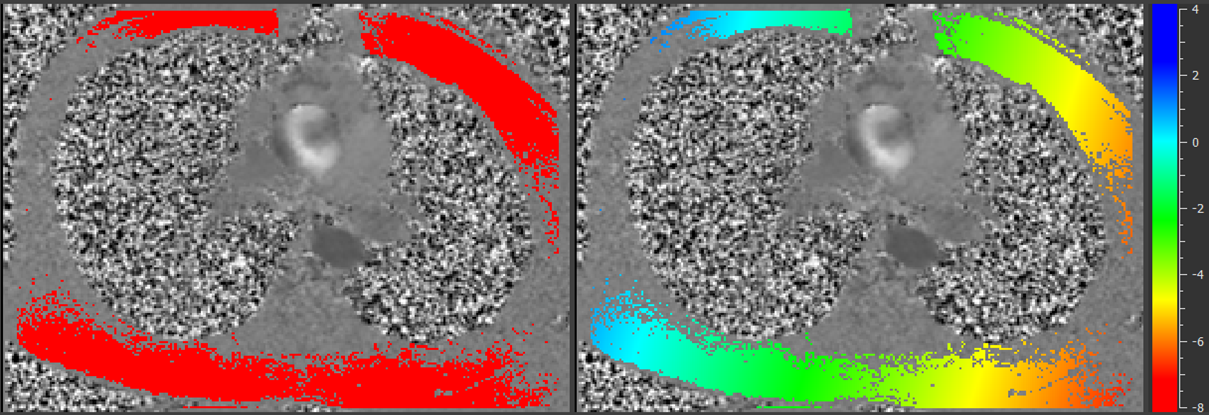

Supplement: Supplementary file 1 — Supplementary material 1 (DOC 661 kb) [file 10554_2015_670_MOESM1_ESM.doc]
